# An epigenome-wide association study of insulin resistance in African Americans

**DOI:** 10.1186/s13148-022-01309-4

**Published:** 2022-07-14

**Authors:** Felix P. Chilunga, Karlijn A. C. Meeks, Peter Henneman, Charles Agyemang, Ayo P. Doumatey, Charles N. Rotimi, Adebowale A. Adeyemo

**Affiliations:** 1 Department of Public & Occupational Health, Amsterdam Public Health Research Institute, Amsterdam University Medical Centers, University of Amsterdam, Amsterdam, The Netherlands; 2 Center for Research on Genomics and Global Health, National Human Genome Research Institute, National Institutes of Health, Bethesda, MD USA; 3 Department of Human Genetics, Amsterdam Reproduction & Development Research Institute, Amsterdam University Medical Centers, University of Amsterdam, Amsterdam, The Netherlands

**Keywords:** Insulin resistance, DNA methylation, Type 2 diabetes, African Americans

## Abstract

**Background:**

African Americans have a high risk for type 2 diabetes (T2D) and insulin resistance. Studies among other population groups have identified DNA methylation loci associated with insulin resistance, but data in African Americans are lacking. Using DNA methylation profiles of blood samples obtained from the Illumina Infinium® HumanMethylation450 BeadChip, we performed an epigenome-wide association study to identify DNA methylation loci associated with insulin resistance among 136 non-diabetic, unrelated African American men (mean age 41.6 years) from the Howard University Family Study.

**Results:**

We identified three differentially methylated positions (DMPs) for homeostatic model assessment of insulin resistance (HOMA-IR) at 5% FDR. One DMP (cg14013695, *HOXA5*) is a known locus among Mexican Americans, while the other two DMPs are novel—cg00456326 (*OSR1;* beta = 0.027) and cg20259981 (*ST18;* beta = 0.010). Although the cg00456326 DMP is novel, the *OSR1* gene has previously been found associated with both insulin resistance and T2D in Europeans. The genes *HOXA5* and *ST18* have been implicated in biological processes relevant to insulin resistance. Differential methylation at the significant *HOXA5* and *OSR1* DMPs is associated with differences in gene expression in the iMETHYL database. Analysis of differentially methylated regions (DMRs) did not identify any epigenome-wide DMRs for HOMA-IR. We tested transferability of HOMA-IR associated DMPs from five previous EWAS in Mexican Americans, Indian Asians, Europeans, and European ancestry Americans. Out of the 730 previously reported HOMA-IR DMPs, 47 (6.4%) were associated with HOMA-IR in this cohort of African Americans.

**Conclusions:**

The findings from our study suggest substantial differences in DNA methylation patterns associated with insulin resistance across populations. Two of the DMPs we identified in African Americans have not been reported in other populations, and we found low transferability of HOMA-IR DMPs reported in other populations in African Americans. More work in African-ancestry populations is needed to confirm our findings as well as functional analyses to understand how such DNA methylation alterations contribute to T2D pathology.

**Supplementary Information:**

The online version contains supplementary material available at 10.1186/s13148-022-01309-4.

## Background

African Americans are disproportionally affected by type 2 diabetes (T2D) [1]. The prevalence of T2D among African Americans is nearly twice that of European–ancestry Americans [1]. Various factors such as socio-economic status, genetic predisposition, environmental triggers, as well as lifestyle factors have been found to contribute to the high risk of T2D among African Americans [2, 3]. However, traditional risk factors such as obesity do not fully explain the elevated risk among African Americans compared with European Americans, and it is estimated that genetic variants only explain 17.5% of the phenotypic variance of T2D [4]. Environmental, lifestyle and social determinants are the major players in ethnic differences in health and a better understanding of the mechanisms by which these factors increase T2D risk in African Americans is needed.

Insulin resistance is a major feature of T2D [5], and African Americans have been reported to have a higher degree of insulin resistance relative to European-ancestry Americans [6]. In addition, differences in insulin resistance have been reported between men and women of various ancestries, which may play a role in sex distinctions in T2D prevalence and phenotypes [7, 8]. Given that the onset of T2D is often preceded by many years of increasing insulin resistance, the direct study of insulin resistance can provide insight into the early phases of T2D pathology. Genetic, environmental, and lifestyle factors influence insulin resistance with the epigenome as a potential interface where these factors converge [9]. Epigenetics, which is the study of heritable yet reversible molecular modifications to DNA without altering the DNA sequence, may thus provide new insights into mechanisms underlying insulin resistance in order to improve our understanding of the pathogenesis of insulin resistance [10]. Furthermore, because of the strong effect of environmental and lifestyle factors on the epigenome, studying epigenetics provides an opportunity to improve our understanding of the social pathways underlying health disparities, and the variable and reversible nature of epigenetics provides opportunities for intervention [11]. The best understood and most studied epigenetic modification is DNA methylation, which can modulate gene expression through the binding of methyl groups to CpG dinucleotides in the DNA.

Previous studies among Europeans, European ancestry Americans, Indian Asians, and Mexican Americans have identified several hundred DNA methylation sites associated with insulin resistance [12–16]. However, data are scarce on the association between DNA methylation and insulin resistance among African Americans. African Americans differ from other African ancestry populations in terms of environmental exposures and the amount of non-African genetic admixture (mainly a European ancestry component which is estimated to range from 10 to 20% on average) [17]. Since DNA methylation can be affected by genetic, environmental as well as lifestyle factors, findings from other population groups cannot be extrapolated to African Americans who have dissimilar genetic variation, environmental exposures, and lifestyles.

Hence, we aimed to identify DNA methylation loci associated with insulin resistance among African American men, using data from the Howard University Family Study (HUFS) [18]. Studying DNA methylation changes associated with insulin resistance may contribute to identifying markers of early pathological changes related to T2D.

## Results

### Participant characteristics

Characteristics of the 136 unrelated African American men without T2D are shown in Table 1. Almost 80% of the participants consumed alcohol, and 73% were current smokers. The mean BMI was 27.2 kg/m^2^, while their glycolytic markers, such as fasting glucose and insulin levels, were in the normal range. Granulocytes represented about 50% of the immune cells inferred by Houseman et al. [19] and each of the other immune cell types was less than 10% as shown in Table 1. The immune cells showed low correlation with insulin resistance assessed using Homeostatic Model Assessment (HOMA-IR) with an r^2^ ranging from 0.057 for CD8 + to 0.204 for CD4 + .

**Table 1:** Participant characteristics (*n* = 136)

	Mean (SD)
Demographics	
Age (years)	41.6 (10.0)
Health-related behavior factors	
Smoking, n (%)	
Never	9 (6.6)
Current	100 (73.5)
Former	27 (19.9)
Alcohol consumption, n (%)	
No	28 (20.6)
Yes	108 (79.4)
Body Mass Index (BMI), kg/m^2^	27.2 (6.1)
Obesity (BMI ≥ 30), n (%)	40 (29.4)
Blood samples	
Fasting glucose (mmol/L)	4.8 (0.6)
Insulin (pmol)	80.5 (44.6)
HOMA-IR	1.19 (1.13)
Immune cells (%)	
CD4 + cells	18.0 (6.4)
CD8 + cells	8.7 (7.1)
Natural killer cells	6.1 (4.5)
B cells	7.9 (3.6)
Monocytes	9.4 (3.1)
Granulocytes	49.9 (11.5)

*SD* standard deviation, *HOMA-IR* homeostatic model assessment for insulin resistance

### Association between DNA methylation and HOMA-IR

**Fig. 1:**
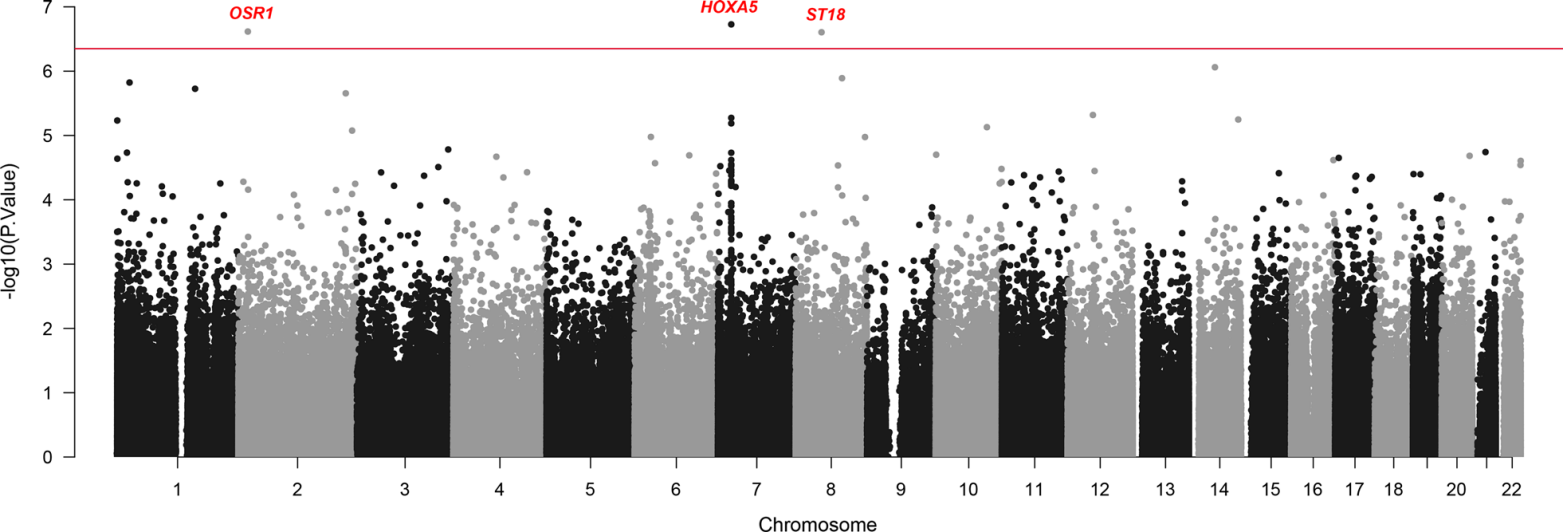
Manhattan plot of epigenome-wide P-values for HOMA-IR in African American men. The red line indicates FDR < 0.05

*Differentially methylated positions (DMPs):* Epigenome-wide DMPs for HOMA-IR were identified using linear regression analyses with adjustment for age, alcohol consumption, tobacco smoking, BMI, estimated cell types, and technical effects (hybridization batch and array position). DNA methylation levels of three CpG sites (cg14013695, cg00456326, cg20259981) showed genome-wide significant associations with HOMA-IR at a 5% false discovery rate (FDR) (Table 2; Fig. 1, Additional file 1: Supplementary Fig. S1). Using *GapHunter,* we confirmed that none of these 3 DMPs had an underlying multimodal distribution. cg14013695 was annotated to the transcription start site (TSS1500) of *HOXA5* on chromosome 7. Our results showed that a single unit higher level of HOMA-IR was associated with a 1.6% lower DNA methylation of cg14013695 (FDR = 0.035). The second DMP (cg00456326) was located on chromosome 2 in the TSS1500 of the gene *OSR1* and was within the shores of the nearest CpG island. DNA methylation levels of cg00456326 were 2.7% higher for each unit higher level of HOMA-IR (FDR = 0.035). The third genome-wide significant CpG site (cg20259981) was annotated to the 5’UTR of *ST18* on chromosome 8 in the open sea. One-unit higher HOMA-IR was associated with 1% lower DNA methylation of cg20259981.

**Table 2:** Top 10 DMPs associated with insulin resistance in African American men

No	CpG ID	Chr	Position	Gene name^a^	Feature^a^	Relation to Island^a^	Delta *β* value	*P*. value	FDR^f^
1	cg14013695	7	27,184,176	*HOXA5*	TSS1500^b^	Island	− 0.016	1.87e − 07	0.035
2	cg00456326	2	19,560,467	*OSR1*	TSS1500^b^	N_Shore	0.027	2.42e − 07	0.035
3	cg20259981	8	53,301,664	*ST18*	5'UTR^c^	OpenSea	− 0.010	2.48e − 07	0.035
4	cg14364984	14	53,310,508	*Intergenic*	*Intergenic*^*d*^	OpenSea	− 0.014	8.68e − 07	0.093
5	cg22885024	8	95,274,933	*GEM*	TSS1500^b^	S_Shore	− 0.005	1.28e − 06	0.107
6	cg10584797	1	26,126,588	*SEPN1*	TSS200^e^	OpenSea	0.002	1.50e − 06	0.107
7	cg18886071	1	160,617,057	*SLAMF1*	5'UTR^c^	OpenSea	0.023	1.87e − 06	0.115
8	cg13437337	2	220,300,109	*SPEG*	Body	Island	0.002	2.21e − 06	0.118
9	cg10170677	12	51,985,615	*SCN8A*	5'UTR^c^	S_Shore	0.002	4.80e − 06	0.209
10	cg20817131	7	27,184,167	*HOXA5*	TSS1500^b^	Island	− 0.026	5.32e − 06	0.209

^a^Annotation was performed via IlluminaHumanMethylation450kanno.ilmn12.hg19. Homo sapiens (human) genome assembly GRCh37 (hg19). Hansen [20] IlluminaHumanMethylation450kanno.ilmn12.hg19: Annotation for Illumina's 450 k methylation arrays. R package version 0.6.0

^b^TSS1500—transcription start site 1500 (the region from Transcription start site (TSS) to − 1500 nucleotides upstream of TSS)

^c^5’UTR—5′ untranslated region (the region of an mRNA that is directly upstream from the initiation codon)

^d^CpG is located approximately 25 kilobases (kb) downstream of FERMT2 gene

^e^TSS200—transcription start site 200 (the region from Transcription start site (TSS) to − 200 nucleotides upstream of TSS)

^f^FDR = false discovery rate (a 5% FDR is considered significant)

**Fig. 2:**
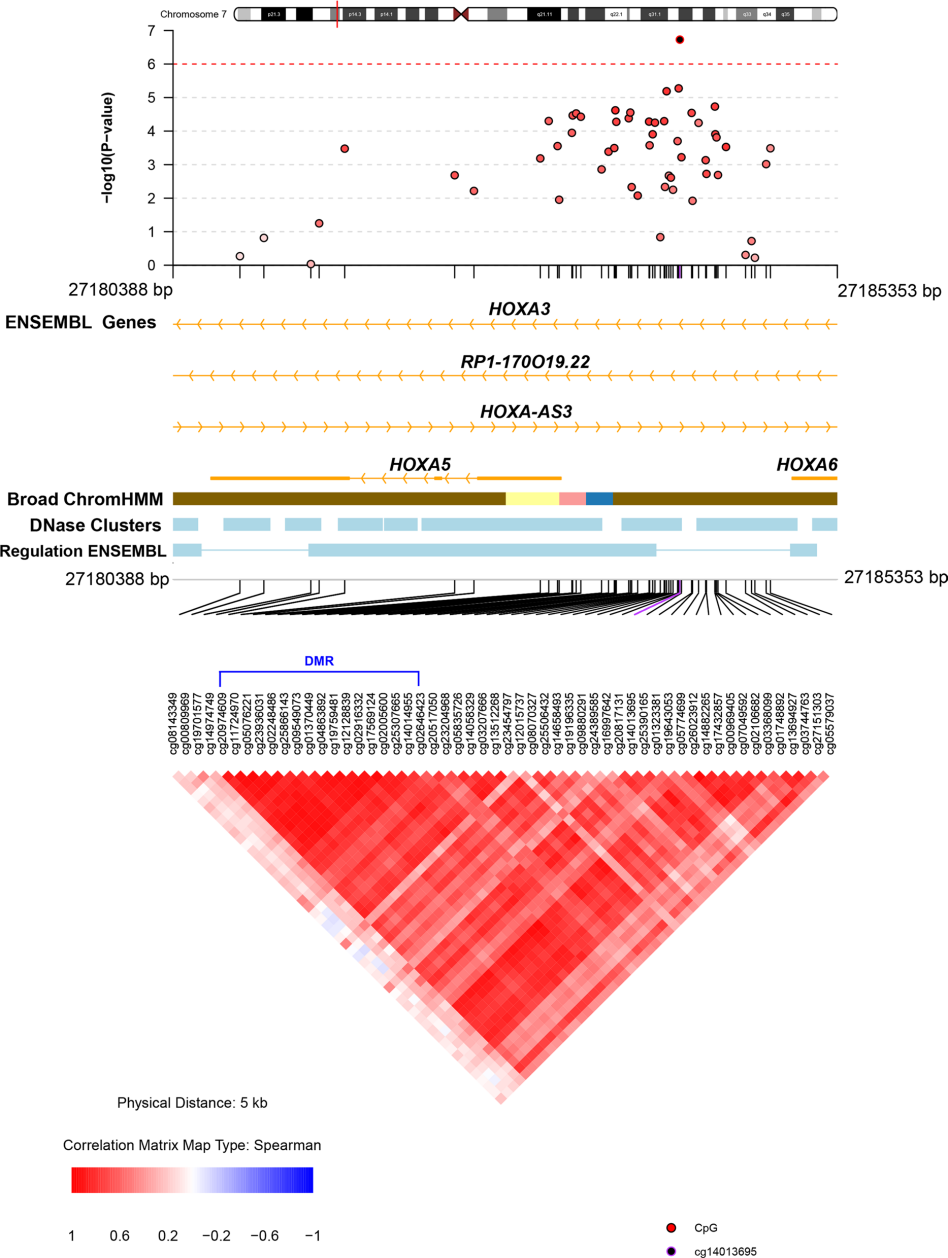
Differentially methylated region for insulin resistance in African Americans annotated to the *HOXA5* gene. Beta values for CpG sites in the DMR are provided in Additional file 2: Supplementary Table S2

*Differentially methylated regions (DMRs):* Our study did not identify any DMRs, defined as three or more CpG sites within the direct vicinity of each other, at a family-wise error rate (FWER) of < 0.05 or FDR < 0.05 using the *Bumphunter* and *DMRcate* packages, respectively. However, our topmost DMR identified with *Bumphunter* (FWER = 0.124) was annotated to the TSS1500 of *HOXA5* on chromosome 7, a regulatory region from which our topmost DMP (cg14013695) was also identified. The DMP cg14013695 was situated 900 bp downstream of the DMR, and although cg14013695 was not among the 17 CpG sites that this DMR consisted of the direction of effect was the same. The DMP as well as the 17 CpG sites of the DMR were hypomethylated for each unit increase in HOMA-IR (Table 3, Fig. 2, Additional file 2: Supplementary Table S1).

**Table 3:** Top 5 differentially methylated regions associated with insulin resistance in African American men

DMR^a^	Chr^b^	Start^c^	End^c^	Gene name^c^	No of CpG sites	*P*-value	FWER^d^	Direction of effect
1	7	27,182,493	27,183,806	*HOXA5*	17	2.4e − 05	0.124	hypomethylated
2	17	79,905,236	79,905,263	*MYADML2*	3	1.9e − 04	0.553	hypomethylated
3	17	41,277,974	41,278,380	*BRCA1*	10	2.1e − 04	0.683	hypomethylated
4	16	53,407,013	53,407,808	*CHD9*	7	2.6e − 04	0.703	hypomethylated
5	22	24,384,105	24,384,400	*GSTT1*	10	2.7e − 04	0.766	hypomethylated

^a^DMR = Differentially methylated region

^b^Chr = Chromosome

^c^Annotation was performed via IlluminaHumanMethylation450kanno.ilmn12.hg19. Homo sapiens (human) genome assembly GRCh37 (hg19). Hansen [20] IlluminaHumanMethylation450kanno.ilmn12.hg19: Annotation for Illumina's 450 k methylation arrays. R package version 0.6.0

^d^FWER = family-wise error rate (an FWER < 0.05 is considered significant)

### Evaluation of our top DMPs in previous EWAS

We searched the literature to determine if our three genome-wide significant DMPs have been reported in previous EWAS. We found that cg14013695 (TSS1500 of *HOXA5*) was previously reported in an EWAS on insulin resistance among Mexican Americans (Beta = − 0.174, *p* = 1.49E − 7, FDR = 0.056) [9]. The same DMP has also been reported in relation to oral squamous cell carcinoma, colorectal cancer, and inflammatory bowel disease among others [21]. The other two DMPs -cg00456326 in the TSS1500 of *OSR1* and cg20259981 in the TSS1500 of *ST18*- have been associated with atherosclerosis and Down syndrome and with asthma, respectively. Next, we searched whether genes annotated to our top three DMPs (i.e., *HOXA5, OSR1, and ST18*) had been reported in previous EWAS. CpG sites annotated to the *HOXA5* gene have been associated to 33 different traits, with the most frequently reported associations including depressive disorders (46 associations), Kabuki syndrome (33 associations), and inflammatory bowel disease (23 associations) [21]. The most-reported associations for CpG sites within the *OSR1* gene are for breast cancer (6 associations), and atherosclerosis (5 associations). Down syndrome (11 associations) and asthma (7 associations) are most reported for the *ST18* gene. With respect to cardiometabolic traits, we found that *HOXA5* was reported in an EWAS on obesity in African Americans, while *OSR1* was reported in an EWAS on insulin resistance and T2D among European populations [3, 12, 22].

### Transferability analysis of CpG sites identified in other populations

We tested the transferability of a total of 855 CpG sites reported in five previous EWAS in Mexican Americans, Indian Asians, Europeans, and European ancestry Americans (Additional file 2: Supplementary Tables S2 and S3) [12–16]. Analysis of the previously reported DMPs was performed using linear regression models similar to those employed in the main analysis. A total of 848 unique CpG sites remained after the removal of 7 CpG sites duplicated across studies. Exclusion of an additional 118 probes that did not meet quality-control thresholds in our data resulted in 730 candidate DMPS included in the final linear regression analysis (Additional file 2: Supplementary Table S4). We found that in our sample of African American men, only cg14013695 (TSS1500 of *HOXA5*) was statistically significant at a Bonferroni-corrected *p*-value of 6.8E − 05 (i.e., *p* = 0.05/730; Additional file 2: Supplementary Table S4). We also assessed whether DMPs identified in other populations were statistically significant in our main analyses. We found that 47 (6.4%) DMPs (including cg14013695) were statistically significant at *p* < 0.05 (Additional file 2: Supplementary Tables S5 and S6). The majority of the replicated DMPs (40/47; 93%) were from the study by Apron et al.[12]. While these DMPs reached significance in the replication, we were not able to check for consistency in direction of effect for many DMPs as effect sizes were not reported for these [12]. However, for the three DMPs whose effect sizes were reported (cg14013695, cg17126947, cg22065733), the direction of effect was consistent.

### Biological pathways and chromatin state enrichment

We conducted enrichment analyses using the *clusterProfiler* package and the EWAS Toolkit in order to assess whether our top 100 DMPs enriched to particular biological pathways at a 5% FDR. The *clusterProfiler* Gene Ontology (GO) results showed that CpG sites annotated to *HOXA5, OSR1, HOXA1, PAX8, HIPK1*, and *HOXA6* were enriched to embryonic organ morphogenesis and embryonic organ development (FDR = 0.030). The *clusterProfiler* KEGG pathway analysis did not yield any enriched pathways. Furthermore, *clusterProfiler* enrichment analysis for chromatin state, CpG Islands and gene position showed that top DMPs were enriched to the flanking active TSSs (*P*-value = 0.026) and enhancers (*P*-value = 0.002), were within CpG Islands (*P*-value = 0.03) and were within the TSS1500 (*P*-value = 0.05) (Additional file 2: Supplementary Table S7a). Enrichment analysis using the EWAS Toolkit did not reveal any GO or KEGG enriched pathways at an FDR < 0.05. The EWAS Toolkit chromatin state analysis showed enrichment to the flaking active TSSs and enhancers in white blood cells and skeletal muscle cells (Additional file 2: Supplementary Table S7b).

### DNA methylation at top loci and gene expression

In order to gain insight into the relationship between DNA methylation differences and gene expression, we assessed the correlation between our top three DMPs and gene expression in white blood cells using the IMETHYL database [23]. We found that high DNA methylation levels of cg14013695 (TSS1500 of *HOXA5*) were associated with low expression of *HOXA5* (Fragments Per Kilobase of transcript per Million mapped reads; FPKM = − 0.11 ± 0.39). Low DNA methylation of cg00456326 (TSS1500 of *OSR1*) was associated with decreased expression of *OSR1* (FPKM = − 0.64 ± 0.18). Such data for cg20259981 (TSS1500 of *ST18*) were not available in the IMETHYL database (Table 4). Querying the EWAS Toolkit showed that cg14013695 was positively correlated with *HOXA5* expression in liver (*R*^2^ = 0.332, *P*-value = 1.32E − 11) and kidney (*R*^2^ = 0.124, *P*-value = 0.001) tissue and that cg20259981 was positively correlated with expression of *ST18* in brain (*R*^2^ = 0.119, *P*-value = 0.004) and testis tissue (*R*^2^ = 0.434, *P*-value = 1.48E − 7).

**Table 4:** Relationship between DNA methylation and gene expression as reported in the IMETHYL database

CpG ID	Nearest gene^a^	Gene feature^a^	Methylation level^b^	Methylation average^b^ % (SD)	FPKM^b,c^ average (SD)
cg14013695	*HOXA5*	TSS1500	High	85.3 (11.2)	− 0.11 (0.39)
cg00456326	*OSR1*	TSS1500	Low	35.0 (12.0)	− 0.64 (0.18)
cg20259981	*ST18*	5'UTR	High	94.2 (5.3)	Data not available

^a^Annotation was performed via IlluminaHumanMethylation450kanno.ilmn12.hg19. Homo sapiens (human) genome assembly GRCh37 (hg19). Hansen [20] IlluminaHumanMethylation450kanno.ilmn12.hg19: Annotation for Illumina's 450 k methylation arrays. R package version 0.6.0

^b^Methylation level according to iMETHYL database (low, medium, high). IMETHYL provides whole-DNA methylation (~ 24 million autosomal CpG sites), whole-genome (~ 9 million single-nucleotide variants), and whole-transcriptome (> 14 000 genes) data for CD4^+^ T-lymphocytes, monocytes, and neutrophils collected from approximately 100 subjects. Komaki et al. [23] iMETHYL: an integrative database of human DNA methylation, gene expression, and genomic variation. Hum Genome Var 5, 18,008 (2018)

^c^FPKM = Fragments Per Kilobase of transcript per Million mapped reads

### Assessment of gene function related to insulin resistance

We assessed whether genes annotated to our top three DMPs were linked to insulin resistance traits in the GWAS catalog, EWAS catalog, and GeneCards. In the GWAS catalog, we found out that genetic variants annotated to *HOXA5, OSR1* and *ST18* were not independently related to insulin resistance traits (Additional file 2: Supplementary Table S8). However, several genes located in the 500 kilobases (kb) vicinity of *HOXA5 (i.e., HOXA11-AS, HOXA11, HOXA-AS3, HOXA3, HOTTIP, HOXA6)* and *ST18 (i.e., RB1CC1)* were associated with BMI adjusted waist circumference and BMI adjusted waist-to-hip ratio (Additional file 2: Supplementary Table S8). In the EWAS catalog, we found that aberrant DNA methylation in *HOXA5* (including cg14013695) was associated with pancreatic ductal adenocarcinoma and gene expression in the liver (Additional file 2: Supplementary Table S9A). Several genes in the 500 kb vicinity of *HOXA5* (i.e., *HOXA3, HOXA9, HOXA7, HOXA1, EVX1)* were also associated with high density lipoprotein (HDL) cholesterol efflux capacity, BMI, and pancreatic ductal adenocarcinoma (Additional file 2: Supplementary Table S9B). The genes *ST18* and *OSR1,* as well genes in their 500 kb vicinity, were not associated with any insulin resistance traits.

## Discussion

In this EWAS of insulin resistance, we have identified three DMPs (i.e., cg14013695, cg00456326, cg20259981) associated with insulin resistance among African American men at a 5% FDR. Two of these DMPs—i.e., cg00456326 (*OSR1*) and cg20259981 (*ST18*)—have not been reported in other populations. In addition, we successfully replicated another 46 DMPs, which were previously identified in other populations. To our knowledge, this is the first EWAS on insulin resistance among African Americans.

Differential methylation of our top DMP, cg14013695 (*HOXA5*) seems to play a role in the pathogenesis of insulin resistance across populations. This DMP has previously been reported in an insulin resistance EWAS among Mexican Americans [16]. In our sample of African Americans as well as in Mexican Americans, cg14013695 was hypomethylated for higher levels of insulin resistance. The CpG site cg14013695 is located in the transcription start sites of *HOXA5* where lower DNA methylation can increase gene expression [24, 25]. We found that decreased DNA methylation of cg14013695 leads to increased expression of the *HOXA5* gene in blood cells, but to decreased expression in kidney and liver [23]. The *HOXA5* gene located on chromosome 7 is a transcription factor that is involved in regulating human embryonic development and adult stem cell differentiation [26]. More importantly, it has been shown that *HOXA5* is highly expressed in the adipose tissue and plays an active role in regulating adipocyte functions, including differentiation and body fat distribution [27]. Methylation levels of the *HOXA5* promoter region, 600 bp upstream of the transcription start site, were found significantly increased in preadipocytes of first-degree relatives of T2D subjects compared with subjects with no family history of T2D [28]. A similar inverse association between methylation and expression level was found for this promotor region as for the transcription start site in which the cg14013695 is located [28]. Functional studies have further shown that changes in *HOXA5* expression in adipocytes play a role in chronic inflammation through M2 macrophage polarization and the eIF2α/PERK signaling pathways [29]. Besides the functional studies, GWAS have also found associations between genes in the 500 kb vicinity of *HOXA5* (i.e., *HOXA11-AS, HOXA11, HOXA-AS3, HOXA3, HOTTIP, HOXA6*) and BMI adjusted waist-to-hip ratio, as well as BMI-adjusted waist circumference [30]. Likewise, EWAS have also found associations between aberrant DNA methylation in *HOXA5* and pancreatic ductal adenocarcinoma, as well as gene expression in the liver [31]. Moreover, DNA methylation alterations in genes within 500 kb vicinity of *HOXA5 (i.e., HOXA3, HOXA9, HOXA7, HOXA1, EVX1)* have been associated with HDL cholesterol efflux capacity and with BMI [31]. This evidence clearly points to a link between DNA methylation in *HOXA5* and the pathogenesis of insulin resistance. Furthermore, successful replication of this CpG site reported in Mexican Americans in our study could point to the common pathology of insulin resistance between African Americans and Mexican Americans.

The DMP association in the promoter region of *OSR1* (cg00456326) may also play a role in cardiometabolic traits across populations. This DMP has previously been found hypermethylated in postmortem obtained atherosclerotic portions of human aortas compared with donor-matched nonatherosclerotic portions of human aortas from samples in Spain [32]. The CpG site cg00456326 is located in the TSS1500 of *OSR1* where the higher DNA methylation level we observed per unit increase in HOMA-IR is likely to lead to decreased expression of the *OSR1* gene. The *OSR1* gene codes for a transcription factor that plays a role in the regulation of embryonic heart and urogenital development [33]. This gene has also been reported as a tumor suppressor in renal cell carcinomas and gastric cancer [34]. In functional studies, administration of insulin was associated with an increase in phosphorylation of *OSR1*, which in turn increased sodium reabsorption in the kidneys via the Nacl cotransporter (NCC) [35]. Further studies are needed to better understand the potential role of the *OSR1* gene in insulin resistance in African Americans.

Differential methylation of DMP cg20259981 may play a role in insulin action through effects on expression of the *ST18* gene*.* The observed 1% more hypomethylation at the CpG site in the 5’UTR of *ST18* for each unit increase in HOMA-IR can be hypothesized to increase expression of *ST18*, since lower DNA methylation in the gene body usually leads to increased gene expression [24, 25]. The gene *ST18* is a tumor suppressor gene, which was originally characterized as the third member of the neural zinc finger transcription factor family [36]. The *ST18* gene is highly expressed in the pancreatic islets and represents a novel transcriptional mediator of lipotoxicity and cytokine-induced *β*-cell death in the pancreas [36]. Furthermore, *ST18* deletion can significantly reduce cellular insulin levels and increase *β* cell apoptosis [37]. In addition to these functional studies, GWAS have also found associations between genes in the 500 kb vicinity of *ST18* (i.e., *RB1CC1*) and BMI-adjusted waist circumference, as well as BMI-adjusted waist-to-hip ratio.

We also noted in pathway enrichment analyses (gene ontology) that CpG sites annotated to *HOXA1, HOXA5, HOXA6, OSR1, PAX8*, and *HIPK1* enriched to embryonic organ morphogenesis. All six genes are homeobox genes and are directly involved in the formation of many body structures during early embryonic development [38]. Recent work has provided evidence that homeobox genes continue to be regionally expressed in adult tissues [38]. Enrichment of this pathway in our study could point to the fact that DNA methylation is associated with the remodeling of body components (regions/structures) that are critical for insulin resistance during stem cell differentiation. For example, *HOXA5* expression is associated with adipose tissue remodeling [39], while *OSR1* expression is associated with remodeling of sodium transporters in the kidneys [35], and *PAX* expression is associated with enhanced differentiation of insulin-producing cells [40].

We successfully replicated a total of 47 (6.4%) HOMA-IR associated DMPs that were previously identified in other populations. This means that the majority of DMPs reported in other populations did not replicate in our sample of African American men and that transferability of DMPs for insulin resistance across populations is low. This finding corroborates with previous studies which showed that DNA methylation varies strongly by population group/ethnicity due to differences in genetic ancestry and environmental exposures [41]. Given the numerous environmental exposures, including lifestyle factors, that can have a substantial effect on DNA methylation [42], we suspect the lack of transferability to be mainly due to these factors. Hence, DNA methylation findings should not be extrapolated to populations of different ancestry or in different environmental contexts. Importantly, African Americans are not a homogenous group and are exposed to a wide variety of environmental and social determinants, which may affect the epigenome. As such, our study, which is the first on African Americans, is an important first step because it provides the much-needed literature on the African American population, but more EWAS studies among African Americans reflecting diversity in environments and exposures are needed.

The main strengths of this study are that it represents the first EWAS for insulin resistance in African Americans (a previously understudied population) and our top three DMP-IR associations are supported by evidence from GWAS, EWAS, and functional studies. Nonetheless, there are several limitations. Firstly, insulin resistance was calculated using a HOMA calculator, while a hyperinsulinemic euglycemic clamp and intravenous glucose tolerance test are considered the gold standard. Nevertheless, validation studies have found good correlation between euglycemic clamp and HOMA-IR (*r* = 0.8) [43], making it a widely used tool in epidemiologic studies. Secondly, our study had a small sample size and was only conducted in men, which may limit the external validity of the results. However, previous studies have shown sex differences in insulin resistance and distinctions in its effects on CVD risk [7, 8, 44], which highlights the importance of sex-specific analyses. Evidently, there is a need for follow-up studies larger in size that include women-specific analysis. In addition, analysis of sex chromosome DNA methylation patterns in insulin target tissue such as skeletal muscle may provide further insight into T2D pathology given the previously reported chromosome-wide and site-specific differences in DNA methylation on the X chromosome of human pancreatic islets [45]. Thirdly, DNA methylation was measured in blood but preferable tissue for insulin resistance includes adipose tissue and skeletal muscle. Fourth, we did not have data available for replication in an independent sample of African American men and neither were gene expression data available for these participants. However, we used data from the iMethyl database for CpG-expression associations, with the caveat that expression quantitative trait methylation loci (like other quantitative trait loci) may differ between populations. Omics data from diverse populations would provide the ability to identify potential population-specific CpG-expression associations. Lastly, our cross-sectional study design contributes further to precluding the assignment of causality to any CpG site.

## Conclusions

Studying DNA methylation changes associated with insulin resistance can help identify early pathophysiological changes related to prediabetes and T2D. In the present study, we successfully identified three significant HOMA-IR-associated CpGs, one of which has been previously reported and two novel candidate loci that have not been previously reported in other populations. Two of the three loci are implicated in insulin resistance or related traits. More work in African Americans is needed to confirm our findings, as well as functional analyses to understand how such DNA methylation alterations contribute to T2D pathology.

## Methods

### Study design and population

The Howard University Family Study (HUFS) study is a population-based family study that enrolled participants between 2001 and 2008 to investigate the genetic and environmental basis of common complex traits (including hypertension, obesity, diabetes, and other health outcomes among African Americans) [18]. The study did not ascertain families based on any phenotype in order to maximize the utility of the cohort for the study of multiple common traits. The full details of the study have been published elsewhere [18]. In brief, HUFS enrolled a representative sample of African Americans in the Washington, DC metropolitan area. All study enrollees self-identified as African American and were excluded if they were under 14 years of age, pregnant at the time of enrollment or had acute febrile illness or an acute pain episode at the time of clinical examination. For the present study, 144 men who were unrelated and were at least 20 years of age were selected for DNA methylation profiling. After the exclusion of eight individuals with T2D as defined by American Diabetes Association (ADA) criteria, 136 participants remained for the current analysis.

### Phenotypic measurements

The following measurements were obtained through a structured questionnaire: age, sex, alcohol consumption, and tobacco smoking. Alcohol consumption was categorized as any or no consumption. Smoking was categorised into current smokers, past smokers, or non-smokers. Body weight was measured in light clothes on an electronic scale to the nearest 0.1 kg, and height was measured with a stadiometer to the nearest 0.1 cm. Body mass index (BMI) was computed as weight (kg) divided by height squared (m^2^). Fasting plasma glucose concentration was measured using the enzymatic reference method with hexokinase on Roche Cobas Integra 400 Plus or Modular-E analyzers. Fasting insulin was measured by electrochemiluminescence immunoassay (ECLIA) on Roche Modular-E or Elecsys 2010 analyzers (Roche Diagnostics, Indianapolis, IN). Insulin resistance was estimated using the updated Homeostasis Model Assessment (HOMA2), which takes into account variations in hepatic and peripheral glucose resistance, increases in the insulin secretion curve for plasma glucose concentrations above 10 mmol/L (180 mg/dL) and the contribution of circulating proinsulin. Homeostatic Model Assessment for Insulin Resistance (HOMA-IR) calculations were conducted using the University of Oxford HOMA2 calculator (available at: https://www.dtu.ox.ac.uk/homacalculator/).

### DNA methylation processing, profiling, and quality control

Bisulfite treatment of DNA (Zymo EZ DNA MethylationTM kit) was used to deaminate unmethylated cytosine to produce uracil in DNA to conform to the manufacturer’s protocol. The converted DNA was amplified and hybridized on the Infinium® HumanMethylation450 BeadChip which quantifies DNA methylation levels of approximately 485,000 CpG sites. The samples were randomized over two bisulfite conversion and hybridization batches. Raw 450 K data were processed for primary quality control using the statistical software platform “R” (version 3.6.1) and *MethylAid* package (version 1.20). An overview of all “R” packages used in the analyses can be found in Additional file 2: Supplementary Table S10. *MethylAid* detects poor-quality samples using sample-dependent and sample-independent control CpG sites present on the 450 K array itself. *MethylAid* threshold values included methylated and unmethylated intensities of 10.5, overall quality control of 11.75, bisulfite control of 13.25, hybridization control of 12.50 and a detection *p* value of 0.94 [46]. The cluster plots showed all samples were of good quality and none were excluded from further analyses (Additional file 2: Supplementary Fig. S2). We used plots from *Minfi* package to predict sex by clustering samples based on their mean DNA methylation intensities on the X and Y chromosomes. We identified a single sex-discordant sample, which was removed. Quantile normalization was used to normalize the raw 450 K data on the assumption that we would only detect very small DNA methylation changes in association with continuous HOMA-IR values. The *minfi* package was used to remove 17,351 cross-reactive probes and probes containing single nucleotide polymorphisms (SNPs) according to the Illumina 450 k manifest. Removal of these probes resulted in a final set of 456,513 CpG sites that were used in subsequent analysis. Finally, as cell mixture is a source of variability in DNA methylation, white blood cell types were estimated using the method developed by Houseman et al*.* [19] and included as covariates in the analysis.

### Statistical analysis

#### Differentially methylated positions and regions

Statistical analysis was carried out using packages in “R” statistical computing environment [47]. Study population characteristics were presented as proportions for categorical variables and as means (with standard deviations) for normally distributed continuous variables. Linear regressions were conducted to determine associations between DNA methylation and HOMA-IR using the *minfi* package (with DNA methylation are the dependent variable). Age, alcohol consumption, tobacco smoking, BMI, estimated cell types, and technical effects (hybridization batch and array position) were included as covariates. Model fitting was evaluated using a QQ-plot (Additional file 1: Supplementary Fig. S3). For all differentially methylated position (DMP) analyses, M values were calculated as the log2 ratio of the intensities of methylated CpG site versus unmethylated CpG site. Identification of significant DMPs was determined based on P-values corresponding with M values, while beta values were used for visualization [48]. The *GapHunter* function [49] was applied to the top 100 DMPs with the lowest M-value *P*-values to detect potential multimodal distributions. False discovery rate (FDR) was used to correct for multiple testing. A 5% FDR was considered genome-wide significant. Regulatory regions around these DMPs were visualized using the *MEAL* package [50, 51]. To detect differentially methylated regions (DMRs), we tested two different methods. First, we fitted models similar to DMP analyses using the *Bumphunter* function [52] with a cutoff of 0.0151 (which corresponds to 1.5% difference in the Beta-values) and 1000 permutations. Second, the *DMRcate* package was used to detect additional DMRs [53]. Three or more CpG sites within the direct vicinity of each other was considered a DMR. For *Bumphunter*, a family-wise error rate (FWER) < 0.05 was considered statistically significant and for *DMRcate*, an FDR of < 0.05.

#### In silico replication

We conducted in silico replication of previously reported DMPs from five previous EWAS for insulin resistance among Europeans, European ancestry Americans, Indian Asians, and Mexican Americans to test the transferability of these candidate loci in our study. First, we assessed whether our top DMPs were on the list of previously reported DMPs. Second, we assessed whether any of the candidate CpG sites had statistical significance in our study at a nominal *p*-value of 0.05. Lastly, we performed a separate statistical analysis on the candidate loci, employing linear regression methods similar to our main DMP analyses. For this analysis, we assumed statistical significance at a Bonferroni-corrected p-value (0.05/number of candidate loci).

#### Post-omic analyses

We performed several post-omic analyses in order to ascertain biological relevance of our findings. First, we performed pathway enrichment analyses using the *clusterProfiler* package on the top 100 DMPs. We filtered GO and KEGG pathways with a 5% FDR. Second, we performed molecular enrichment analysis for CpG Islands, gene position, and chromatin state separately at an alpha < 0.05. Third, we used the same set of 100 DMPs to perform enrichment analysis with the EWAS Toolkit [21]. Next, we assessed for correlations between top DMPs and gene expression in white blood cells using the publicly available IMETHYL database [23] and the EWAS Toolkit for other tissues [21]. IMETHYL provides whole-DNA, whole-genome, and whole-transcriptome data for normal CD4 + T-lymphocytes, monocytes, and neutrophils collected from approximately 100 healthy subjects [23]. Finally, we searched in the GWAS catalog (https://www.ebi.ac.uk/gwas/), GeneCards (https://www.genecards.org/),the EWAS catalog (http://www.ewascatalog.org/), and EWAS Atlas (https://ngdc.cncb.ac.cn/ewas/atlas) to determine whether genes annotated to top DMPs were linked to insulin resistance.

## Supplementary Information

**Additional file1.** **Figure S1**. Volcano plot of Differentially Methylated Positions (DMPs) for HOMA-IR; **Figure S2**. Quality control plot of the median methylated versus unmethylated signal intensity; **Figure S3.** Q-Q plot of epigenome-wide P-values for HOMA-IR.

**Additional file2.** **Table S1**. List of CpG sites within the HOXA5 DMR; **Table S2**. Previously reported EWAS for insulin resistance; **Table S3**. DMPs reported in previous insulin resistance EWAS; **Table S4**. Association between candidate CpG sites reported in previous studies and HOMA-IR in African American men.**Table S5**. Summary statistics from the main HOMA-IR analysis in African American men for candidate CpG sites; **Table S6**. Candidate probes that successfully replicated in African American men; **Table S7a**. Chromatin states of top 100 DMPs associated with insulin resistance in African American men from clusterProfiler; **Table S7b. **Chromatin states of top 100 DMPs associated with insulin resistance in African American men from the EWAS Toolkit; **Table S8**. Gene function and associated phenotypes in GWAS catalog and GeneCards; **Table S9A**. Traits associated with the three identified DMPs (annotated genes) in the EWAS catalog; **Table S9B**. Traits associated with genes in the 500kb vicinity of HOXA5, OSR1, and ST18 in the EWAS catalog; **Table S10**. R packages used in the analyses.

## Data Availability

The datasets analyzed during the current study are not publicly available due to the consent obtained which does not grant permission for deposition but are available from the corresponding author on reasonable request as permitted by the IRB approval and signed informed consent.

## References

[1] Cheng YJ, Kanaya AM, Araneta MRG, Saydah SH, Kahn HS, Gregg EW (2019). Prevalence of diabetes by race and ethnicity in the United States, 2011–2016. JAMA.

[2] Chatterjee R, Maruthur NM, Edelman D (2015). Novel risk factors for type 2 diabetes in African-Americans. Curr DiabRep.

[3] Demerath EW, Guan W, Grove ML, Aslibekyan S, Mendelson M, Zhou Y-H (2015). Epigenome-wide association study (EWAS) of BMI, BMI change and waist circumference in African American adults identifies multiple replicated loci. Hum Mol Genet.

[4] Ng MC, Shriner D, Chen BH, Li J, Chen W-M, Guo X (2014). Meta-analysis of genome-wide association studies in African Americans provides insights into the genetic architecture of type 2 diabetes. PLoS Genet.

[5] Zaccardi F, Webb DR, Yates T, Davies MJ (2016). Pathophysiology of type 1 and type 2 diabetes mellitus: a 90-year perspective. Postgrad Med J.

[6] Hasson BR, Apovian C, Istfan N (2015). Racial/Ethnic differences in insulin resistance and beta cell function: relationship to racial disparities in type 2 diabetes among African Americans versus Caucasians. Curr Obes Rep.

[7] Goedecke JH, George C, Veras K, Peer N, Lombard C, Victor H (2016). Sex differences in insulin sensitivity and insulin response with increasing age in black South African men and women. Diabetes Res Clin Pract.

[8] Kautzky-Willer A, Brazzale AR, Moro E, Vrbíková J, Bendlova B, Sbrignadello S (2012). Influence of increasing BMI on insulin sensitivity and secretion in normotolerant men and women of a wide age span. Obesity (Silver Spring, Md).

[9] Maude H, Sanchez-Cabanillas C, Cebola I (2021). Epigenetics of hepatic insulin resistance. Front Endocrinol.

[10] Ahmed SAH, Ansari SA, Mensah-Brown EPK, Emerald BS (2020). The role of DNA methylation in the pathogenesis of type 2 diabetes mellitus. Clin Epigenet.

[11] Meloni M, Moll T, Issaka A, Kuzawa CW (2022). A biosocial return to race? A cautionary view for the postgenomic era. Am J Hum Biol.

[12] Arpon A, Milagro FI, Ramos-Lopez O, Mansego ML, Santos JL, Riezu-Boj J-I (2019). Epigenome-wide association study in peripheral white blood cells involving insulin resistance. Sci Rep.

[13] Chambers JC, Loh M, Lehne B, Drong A, Kriebel J, Motta V (2015). Epigenome-wide association of DNA methylation markers in peripheral blood from Indian Asians and Europeans with incident type 2 diabetes: a nested case-control study. Lancet Diabetes Endocrinol.

[14] Hidalgo B, Irvin MR, Sha J, Zhi D, Aslibekyan S, Absher D (2014). Epigenome-wide association study of fasting measures of glucose, insulin, and HOMA-IR in the Genetics of Lipid Lowering Drugs and Diet Network study. Diabetes.

[15] Kriebel J, Herder C, Rathmann W, Wahl S, Kunze S, Molnos S (2016). Association between DNA methylation in whole blood and measures of glucose metabolism: KORA F4 study. PLoS One.

[16] Kulkarni H, Kos MZ, Neary J, Dyer TD, Kent JW, Göring HH (2015). Novel epigenetic determinants of type 2 diabetes in Mexican-American families. Hum Mol Genet.

[17] Zakharia F, Basu A, Absher D, Assimes TL, Go AS, Hlatky MA (2009). Characterizing the admixed African ancestry of African Americans. Genome Biol.

[18] Adeyemo A, Gerry N, Chen G, Herbert A, Doumatey A, Huang H (2009). A genome-wide association study of hypertension and blood pressure in African Americans. PLoS Genet.

[19] Houseman EA, Kelsey KT, Wiencke JK, Marsit CJ (2015). Cell-composition effects in the analysis of DNA methylation array data: a mathematical perspective. BMC Bioinform.

[20] Hansen KD. IlluminaHumanMethylation450kanno.ilmn12.hg19: Annotation for Illumina's 450k methylation arrays. R package version 0.6.0. 2016. https://bioconductor.org/packages/release/data/annotation/html/IlluminaHumanMethylation450kanno.ilmn12.hg19.html.

[21] Xiong Z, Yang F, Li M, Ma Y, Zhao W, Wang G (2022). EWAS Open Platform: integrated data, knowledge and toolkit for epigenome-wide association study. Nucleic Acids Res.

[22] Xu X, Su S, Barnes VA, De Miguel C, Pollock J, Ownby D (2013). A genome-wide methylation study on obesity: differential variability and differential methylation. Epigenetics.

[23] Komaki S, Shiwa Y, Furukawa R, Hachiya T, Ohmomo H, Otomo R (2018). iMETHYL: an integrative database of human DNA methylation, gene expression, and genomic variation. Hum Genome Var.

[24] Anastasiadi D, Esteve-Codina A, Piferrer F (2018). Consistent inverse correlation between DNA methylation of the first intron and gene expression across tissues and species. Epigenet Chromatin.

[25] Razin A, Cedar H (1991). DNA methylation and gene expression. Microbiol Rev.

[26] Jeannotte L, Gotti F, Landry-Truchon K (2016). Hoxa5: a key player in development and disease. J Dev Biol.

[27] Parrillo L, Costa V, Raciti G, Longo M, Spinelli R, Esposito R (2016). Hoxa5 undergoes dynamic DNA methylation and transcriptional repression in the adipose tissue of mice exposed to high-fat diet. Int J Obes.

[28] Parrillo L, Spinelli R, Costanzo M, Florese P, Cabaro S, Desiderio A (2022). Epigenetic dysregulation of the homeobox A5 (HOXA5) gene associates with subcutaneous adipocyte hypertrophy in human obesity. Cells.

[29] Cao W, Zhang T, Feng R, Xia T, Huang H, Liu C (2019). Hoxa5 alleviates obesity-induced chronic inflammation by reducing ER stress and promoting M2 macrophage polarization in mouse adipose tissue. J Cell Mol Med.

[30] Welter D, MacArthur J, Morales J, Burdett T, Hall P, Junkins H (2014). The NHGRI GWAS Catalog, a curated resource of SNP-trait associations. Nucleic Acids Res.

[31] Battram T, Yousefi P, Crawford G, Prince C, Babei MS, Sharp G, et al. The EWAS catalog: a database of epigenome-wide association studies. Wellcome Open Res. 2022;7:41. 10.12688/wellcomeopenres.17598.1 PMC9096146 35592546

[32] Zaina S, Heyn H, Carmona FJ, Varol N, Sayols S, Condom E (2014). DNA methylation map of human atherosclerosis. Circ Cardiovasc Genet.

[33] Wang Q, Lan Y, Cho E-S, Maltby KM, Jiang R (2005). Odd-skipped related 1 (Odd1) is an essential regulator of heart and urogenital development. Dev Biol.

[34] Zhang Y, Yuan Y, Liang P, Guo X, Ying Y, Shu X-S (2017). OSR1 is a novel epigenetic silenced tumor suppressor regulating invasion and proliferation in renal cell carcinoma. Oncotarget.

[35] Grimm PR, Taneja TK, Liu J, Coleman R, Chen Y-Y, Delpire E (2012). SPAK isoforms and OSR1 regulate sodium-chloride co-transporters in a nephron-specific manner. J Biol Chem.

[36] Henry C, Close A-F, Buteau J (2014). A critical role for the neural zinc factor ST18 in pancreatic β-cell apoptosis. J Biol Chem.

[37] Cheng C, Lu J, Cao X, Yang F-Y, Liu J-Y, Song L-N (2019). Identification of Rfx6 target genes involved in pancreas development and insulin translation by ChIP-seq. Biochem Biophys Res Commun.

[38] Rux DR, Wellik DM (2017). Hox genes in the adult skeleton: novel functions beyond embryonic development. Dev Dyn.

[39] Cao W, Xu Y, Luo D, Saeed M, Sun C (2018). Hoxa5 promotes adipose differentiation via increasing DNA methylation level and inhibiting PKA/HSL signal pathway in mice. Cell Physiol Biochem.

[40] Lin H-T, Kao C-L, Lee K-H, Chang Y-L, Chiou S-H, Tsai F-T (2007). Enhancement of insulin-producing cell differentiation from embryonic stem cells using pax4-nucleofection method. World J Gastroenterol.

[41] Galanter JM, Gignoux CR, Oh SS, Torgerson D, Pino-Yanes M, Thakur N (2017). Differential methylation between ethnic sub-groups reflects the effect of genetic ancestry and environmental exposures. eLife.

[42] Martin EM, Fry RC (2018). Environmental influences on the epigenome: exposure-associated DNA methylation in human populations. Annu Rev Public Health.

[43] Bonora E, Targher G, Alberiche M, Bonadonna RC, Saggiani F, Zenere MB (2000). Homeostasis model assessment closely mirrors the glucose clamp technique in the assessment of insulin sensitivity: studies in subjects with various degrees of glucose tolerance and insulin sensitivity. Diabetes Care.

[44] Kim SH, Reaven G (2013). Sex differences in insulin resistance and cardiovascular disease risk. J Clin Endocrinol Metab.

[45] Hall E, Volkov P, Dayeh T, Esguerra JLS, Salö S, Eliasson L (2014). Sex differences in the genome-wide DNA methylation pattern and impact on gene expression, microRNA levels and insulin secretion in human pancreatic islets. Genome Biol.

[46] Aryee MJ, Jaffe AE, Corrada-Bravo H, Ladd-Acosta C, Feinberg AP, Hansen KD (2014). Minfi: a flexible and comprehensive Bioconductor package for the analysis of Infinium DNA methylation microarrays. Bioinformatics.

[47] R Core Team. R: A language and environment for statistical computing. In: Computing RFfS, editor. Vienna, Austria, 2021.

[48] Du P, Zhang X, Huang C-C, Jafari N, Kibbe WA, Hou L (2010). Comparison of Beta-value and M-value methods for quantifying methylation levels by microarray analysis. BMC Bioinform.

[49] Andrews SV, Ladd-Acosta C, Feinberg AP, Hansen KD, Fallin MD (2016). “Gap hunting” to characterize clustered probe signals in Illumina methylation array data. Epigenet Chromatin.

[50] Ruiz-Arenas C. MEAL: perform methylation analysis. R package version 1.14.0. 2019.

[51] Ruiz-Arenas C, González JR (2017). Redundancy analysis allows improved detection of methylation changes in large genomic regions. BMC Bioinform.

[52] Jaffe AE, Murakami P, Lee H, Leek JT, Fallin MD, Feinberg AP (2012). Bump hunting to identify differentially methylated regions in epigenetic epidemiology studies. Int J Epidemiol.

[53] Peters TJ, Buckley MJ, Statham AL, Pidsley R, Samaras K, Lord VR (2015). De novo identification of differentially methylated regions in the human genome. Epigenet Chromatin.

