# Supplementary material for: An epigenome-wide association study of insulin resistance in African Americans
Source: Clin Epigenetics. 2022 Jul 14;14:88. doi: 10.1186/s13148-022-01309-4 (PMC9281172; doi:10.1186/s13148-022-01309-4)

SUPPLEMENTARY FIGURES

Figure S1. Volcano plot of Differentially Methylated Positions (DMPs) for HOMA-IR

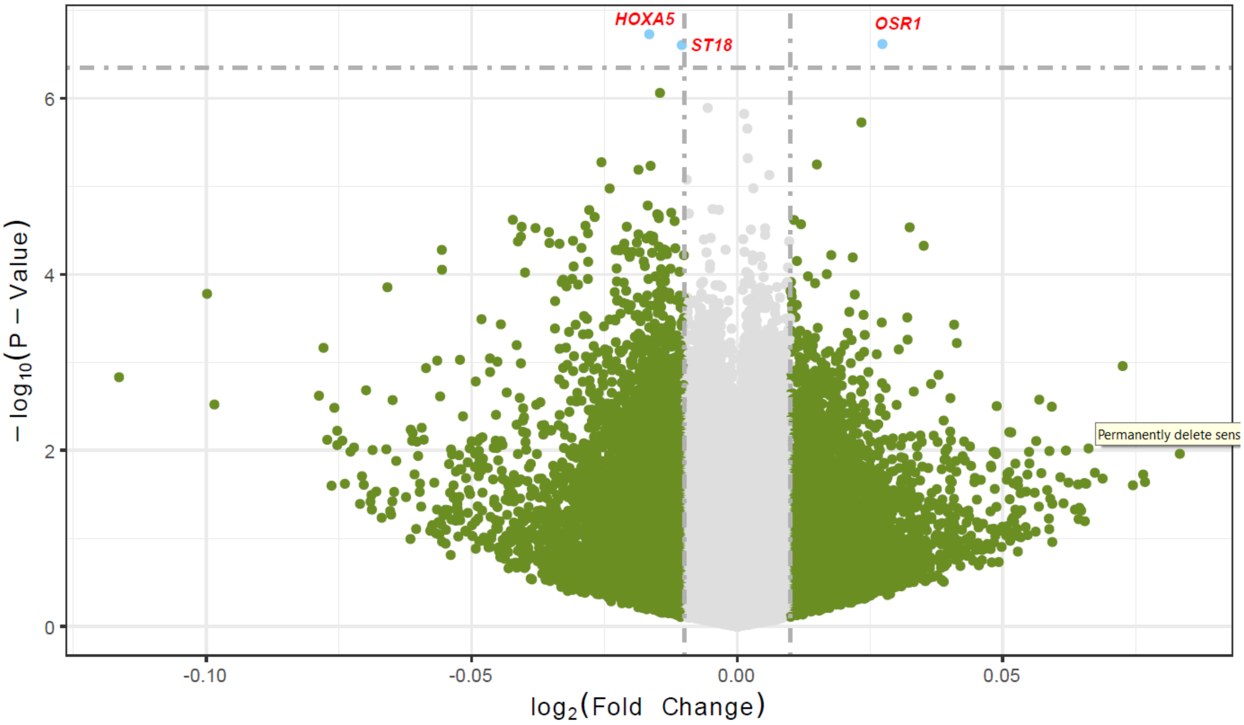

Figure S2. Quality control plot of the median methylated versus unmethylated signal intensity

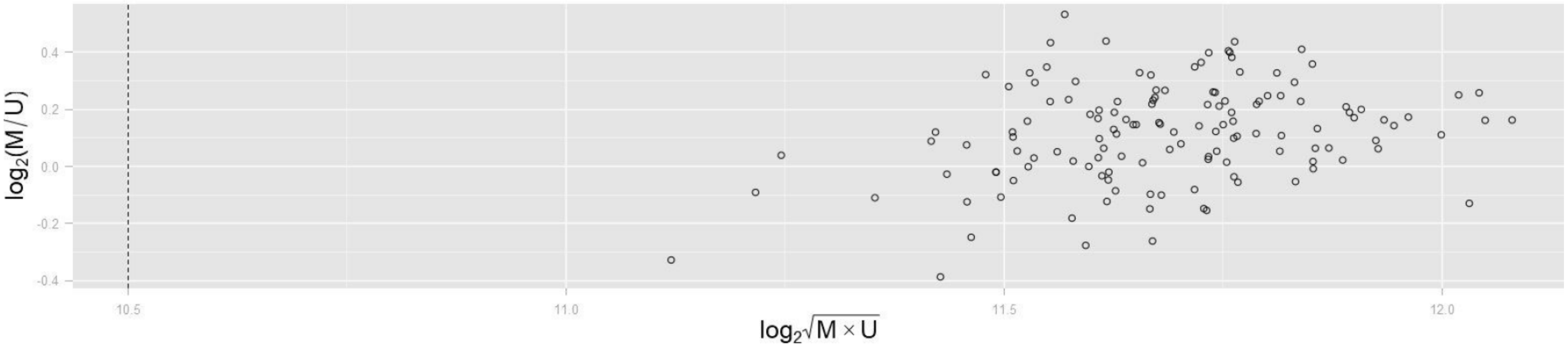

Figure S3. Q-Q plot of epigenome-wide P-values for HOMA-IR

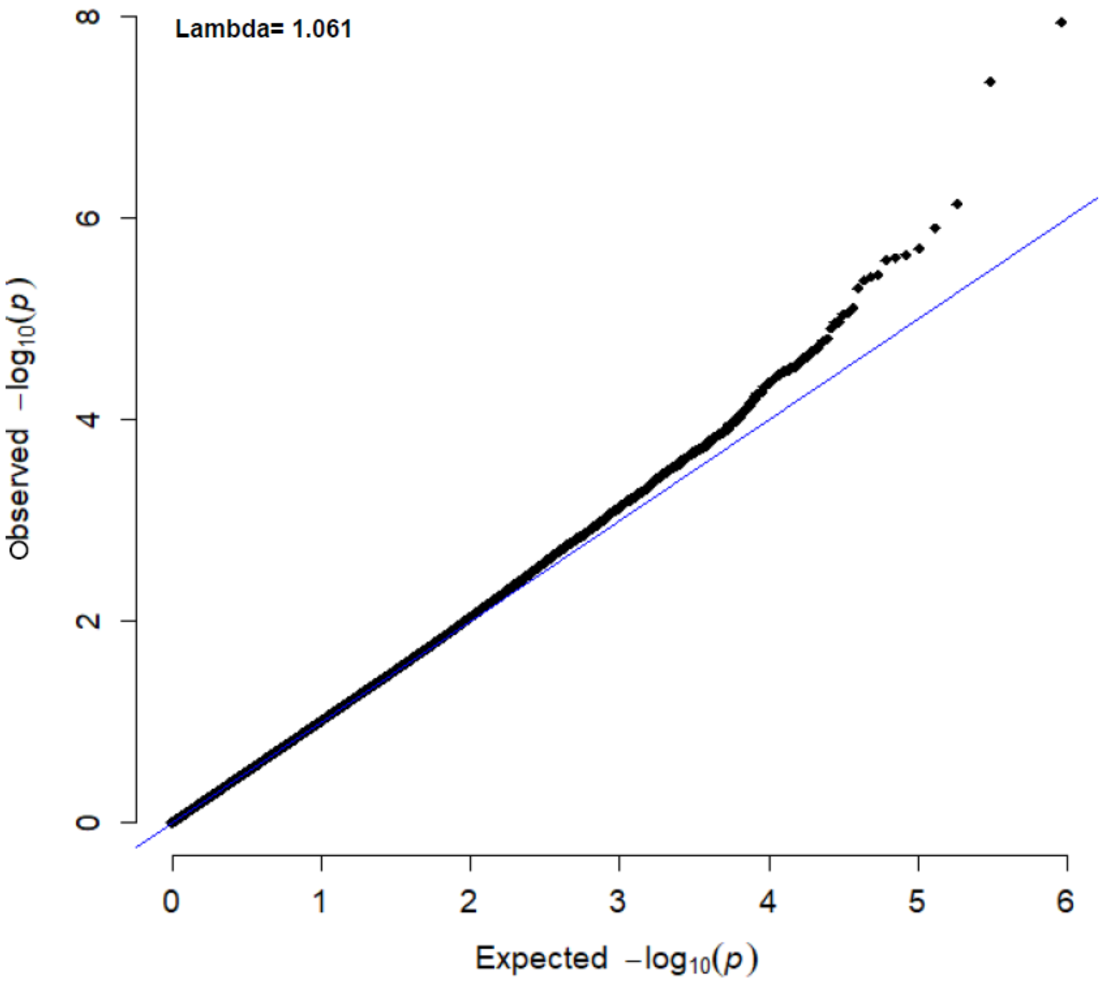

Supplement: Supplementary file 1 — Additional file1. Figure S1. Volcano plot of Differentially Methylated Positions (DMPs) for HOMA-IR; Figure S2. Quality control plot of the median methylated versus unmethylated signal intensity; Figure S3. Q-Q plot of epigenome-wide P-values for HOMA-IR. [file 13148_2022_1309_MOESM1_ESM.pdf]
